# Supplementary material for: Spatial patterns of Holocene temperature changes over mid-latitude Eurasia
Source: Nat Commun. 2024 Feb 19;15:1507. doi: 10.1038/s41467-024-45883-y (PMC10876552; doi:10.1038/s41467-024-45883-y)
Supplement: Supplementary file 1 — Supplementary Information [file 41467_2024_45883_MOESM1_ESM.docx]

**Spatial patterns of Holocene temperature changes** **over mid-latitude Eurasia**

Jiawei Jiang, Bowen Meng, Huanye Wang, Hu Liu, Mu Song, Yuxin He, Cheng Zhao, Jun Cheng, Guoqiang Chu, Sergey Krivonogov, Weiguo Liu, Zhonghui Liu

Correspondence to: ZL ([zhliu@hku.hk](mailto:zhliu@hku.hk)), SK (carpos.sergey@gmail.com), and GC ([chuguoqiang@mail.igcas.ac.cn](mailto:chuguoqiang@mail.igcas.ac.cn))

**Supplementary Fig. 1 Age model for the sediment cores.**

**Supplementary Fig. 2 Alkenone records from mid-latitude Asian lakes.**

**Supplementary Fig. 3 Proportion of alkenone C_37:4_ (%C_37:4_) and hydrological records from marginal monsoon region.**

**Supplementary Fig. 4 Proportion of alkenone C_37:4_ (%C_37:4_) and hydrological records from westerlies region.**

**Supplementary Fig. 5 Alkenone unsaturation (**$\mathbf{U}_{\mathbf{37}}^{\mathbf{K'}}$**) records from mid-latitude Asian lakes.**


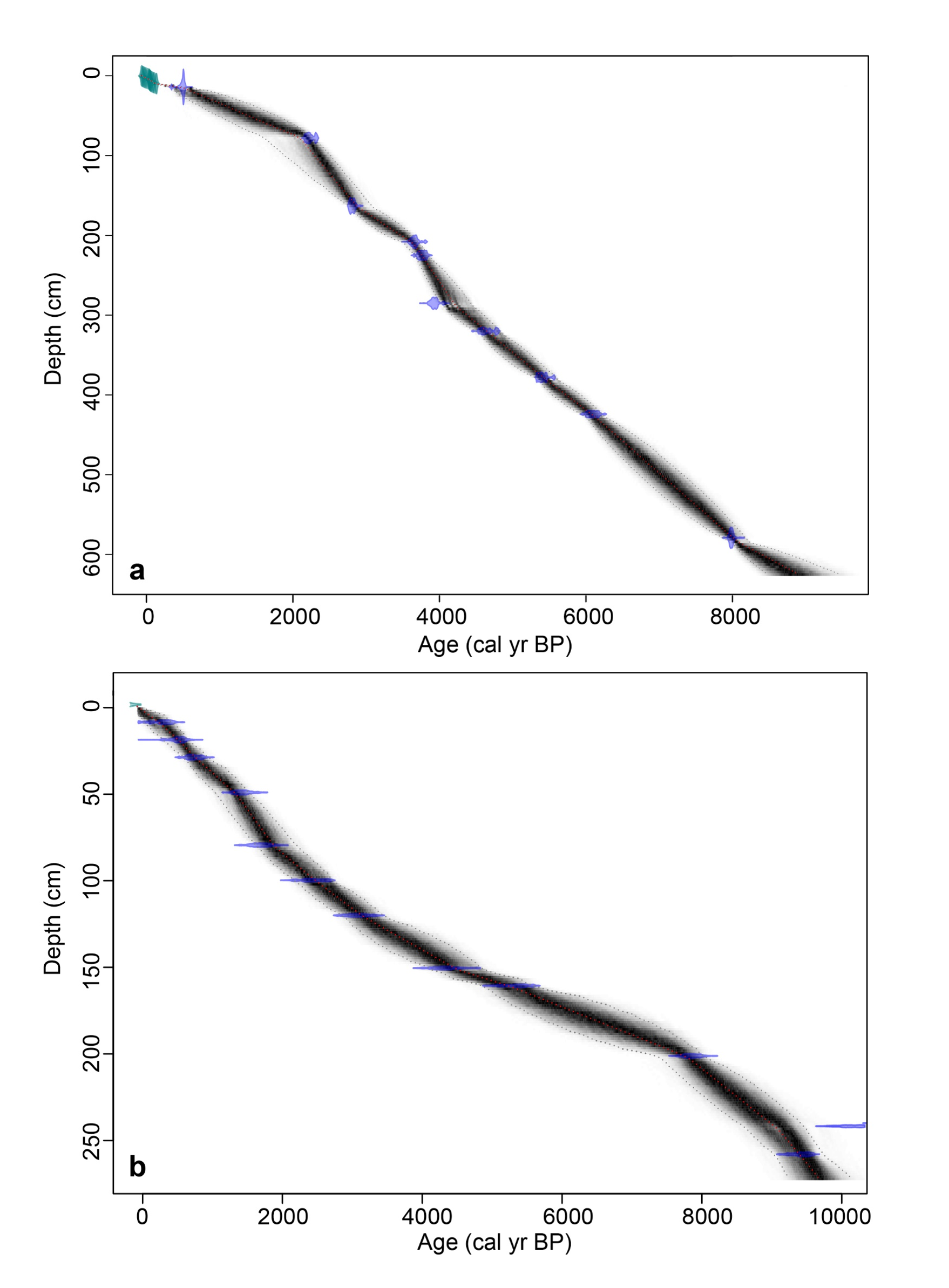


**Supplementary Fig. 1 Age model for the sediment cores.** (**a**) Lake Yihesariwusu, (**b**) Lake Ebeyty**.** The Bayesian program Bacon^65^ was used to fit the age-depth profile to derive the final age model. The shaded area indicates the 95% confidence range and the red dotted line indicates the weighted mean ages for each depth. CRS model ages from Lake Yihesariwusu based on ^210^Pb dates at 0–6.5 cm (green line) have been reported in ref.^63^.


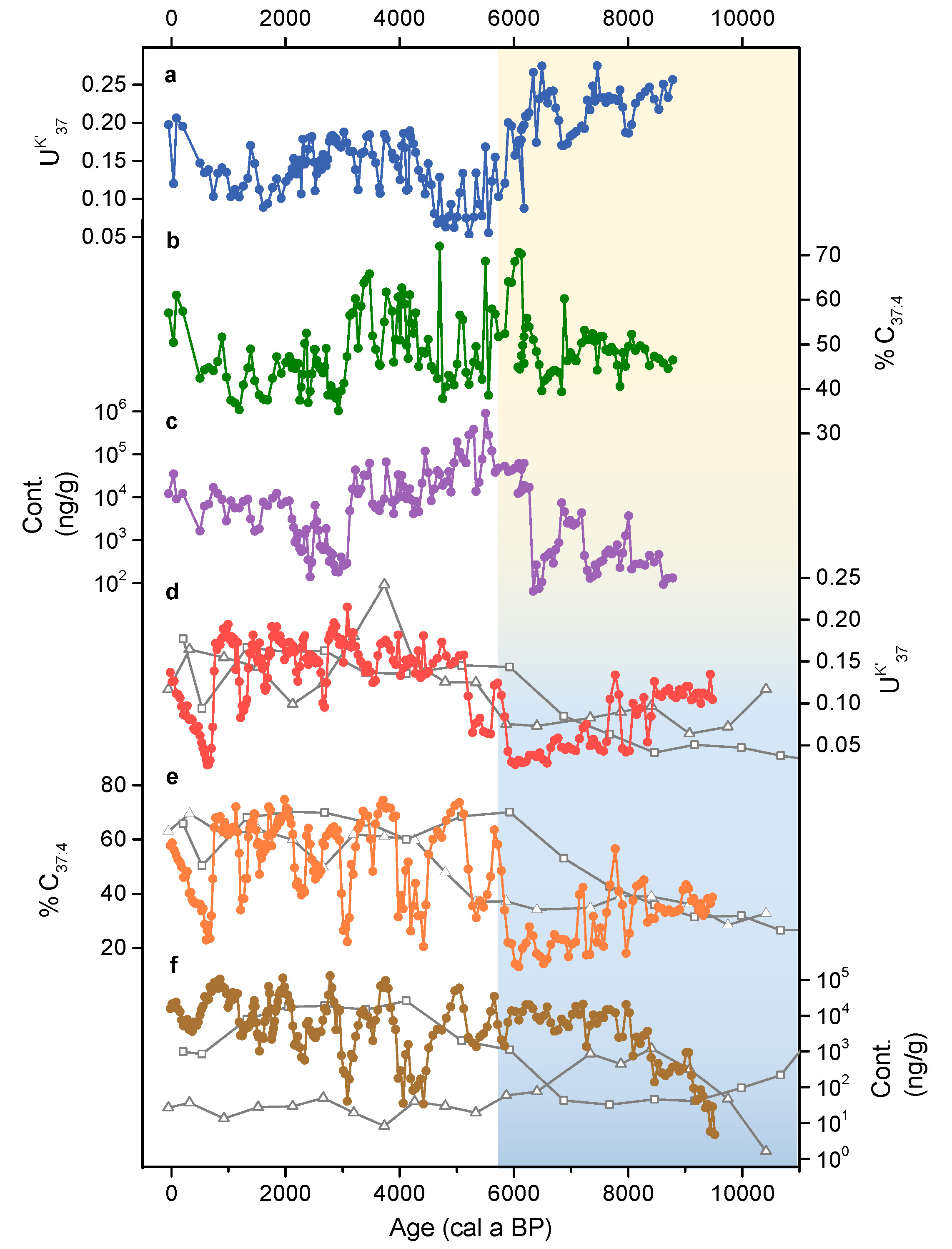


**Supplementary Fig. 2 Alkenone records from mid-latitude Asian lakes.** (**a**) Alkenone unsaturation ($U_{37}^{K'}$) record. (**b**) Proportion of alkenone C_37:4_ (%C_37:4_) record. (**c**) Alkenone C_37_ content from Lake Yihesariwusu, northeastern China. (**d**) $U_{37}^{K'}$, (**e**) %C_37:4_, and (**f**) alkenone C_37_ content from Lake Ebeyty, Kuchuk, and Maloye Yarovoye (dots, squares, and triangles, respectively), southwestern Siberian. Yellow/blue bar indicates warmer/colder conditions during the early to mid-Holocene.


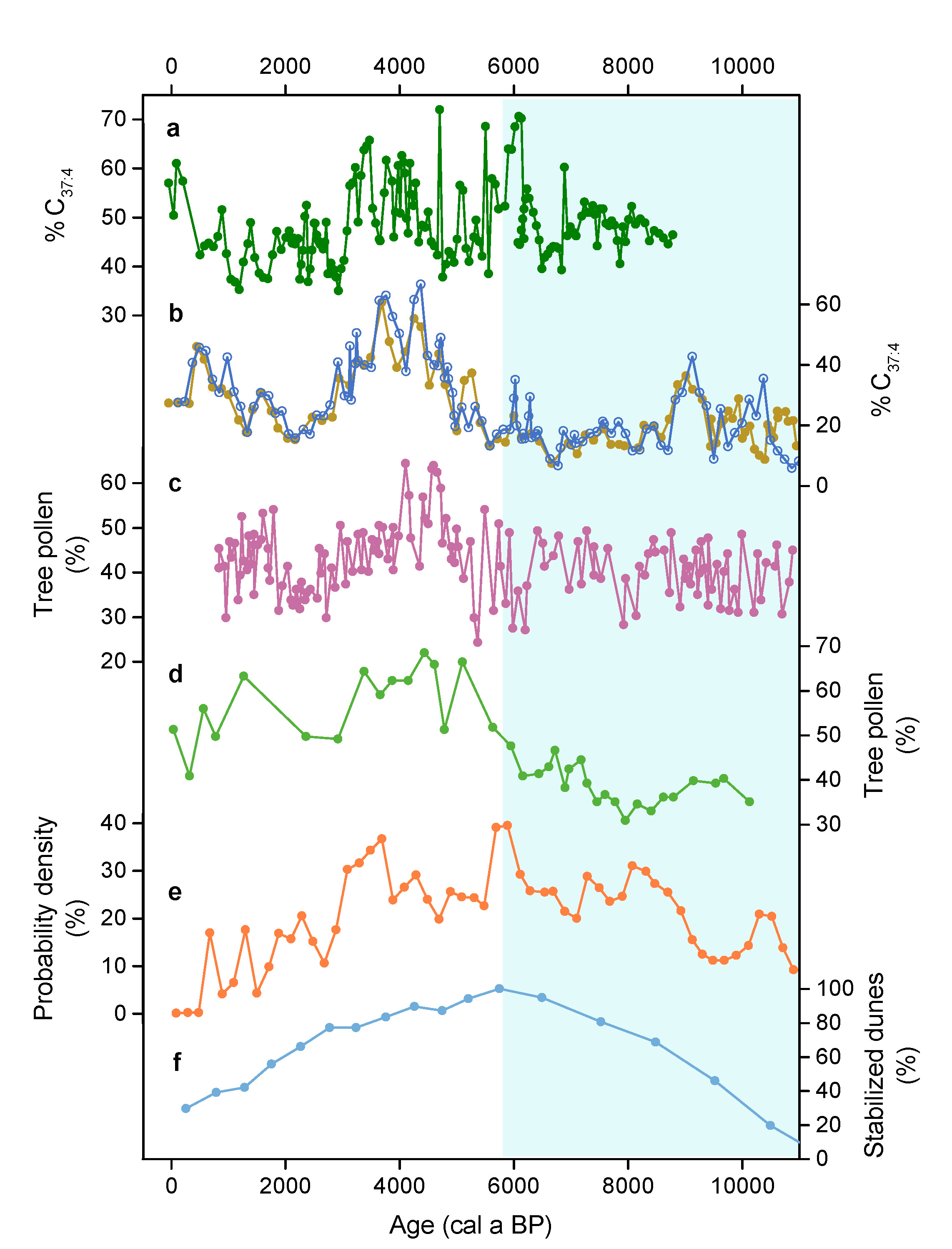


**Supplementary Fig. 3 Proportion of alkenone C_37:4_ (%C_37:4_) and other hydrological records from marginal monsoon region.** %C_37:4_ records from (**a**) Lake Yihesariwusu and (**b**) Lake Qinghai^18,31,32^. Tree pollen percentage records from (**c**) Lake Tianchi^34^ and (**d**) Lake Moon^35^. (**e**) Probability density of paleosol formation on the Chinese Loess Plateau^36^. (**f**) Percentage of stabilized dune sites relative to all sites sampled in the dune fields in northern China^37^. Blue bar highlights the early to mid-Holocene.


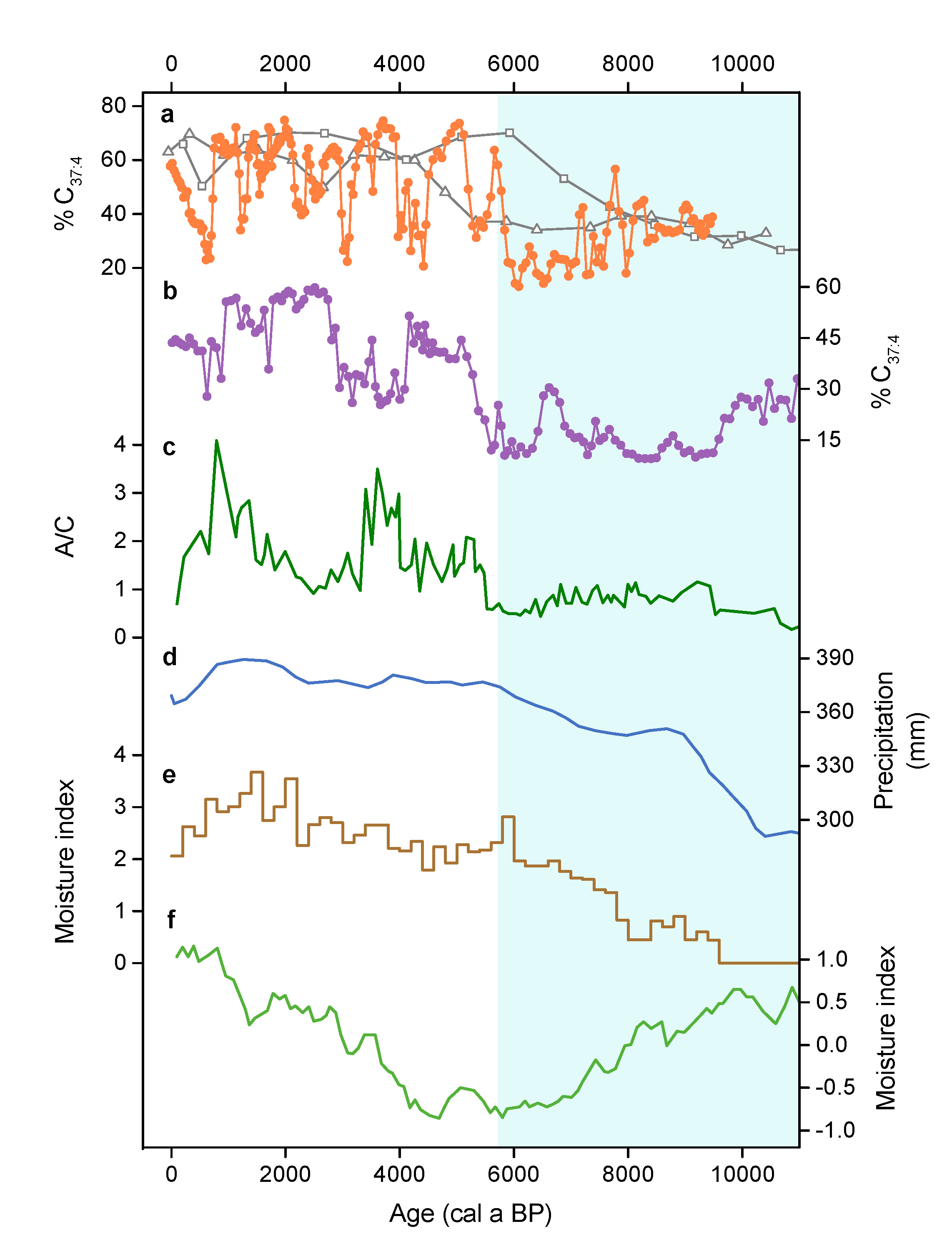


**Supplementary Fig. 4 Proportion of alkenone C_37:4_ (%C_37:4_) and other hydrological records from westerlies region.** %C_37:4_ records from (**a**) Lake Ebeyty, Kuchuk, and Maloye Yarovoye (dots, squares, and triangles, respectively), and (**b**) Lake Sayram^18^. (**c**) Pollen *Artemisia/Chenopodiaceae* (A/C) record from Lake Sayram^38^. (**d**) Pollen-based annual precipitation record from Lake Kuchuk and Maloye Yarovoye^33^. (**e**) Synthesis of moisture records from northern Xinjiang^39^. (**f**) Synthesized moisture index in northern Central Asia^40^. Blue bar highlights the early to mid-Holocene.


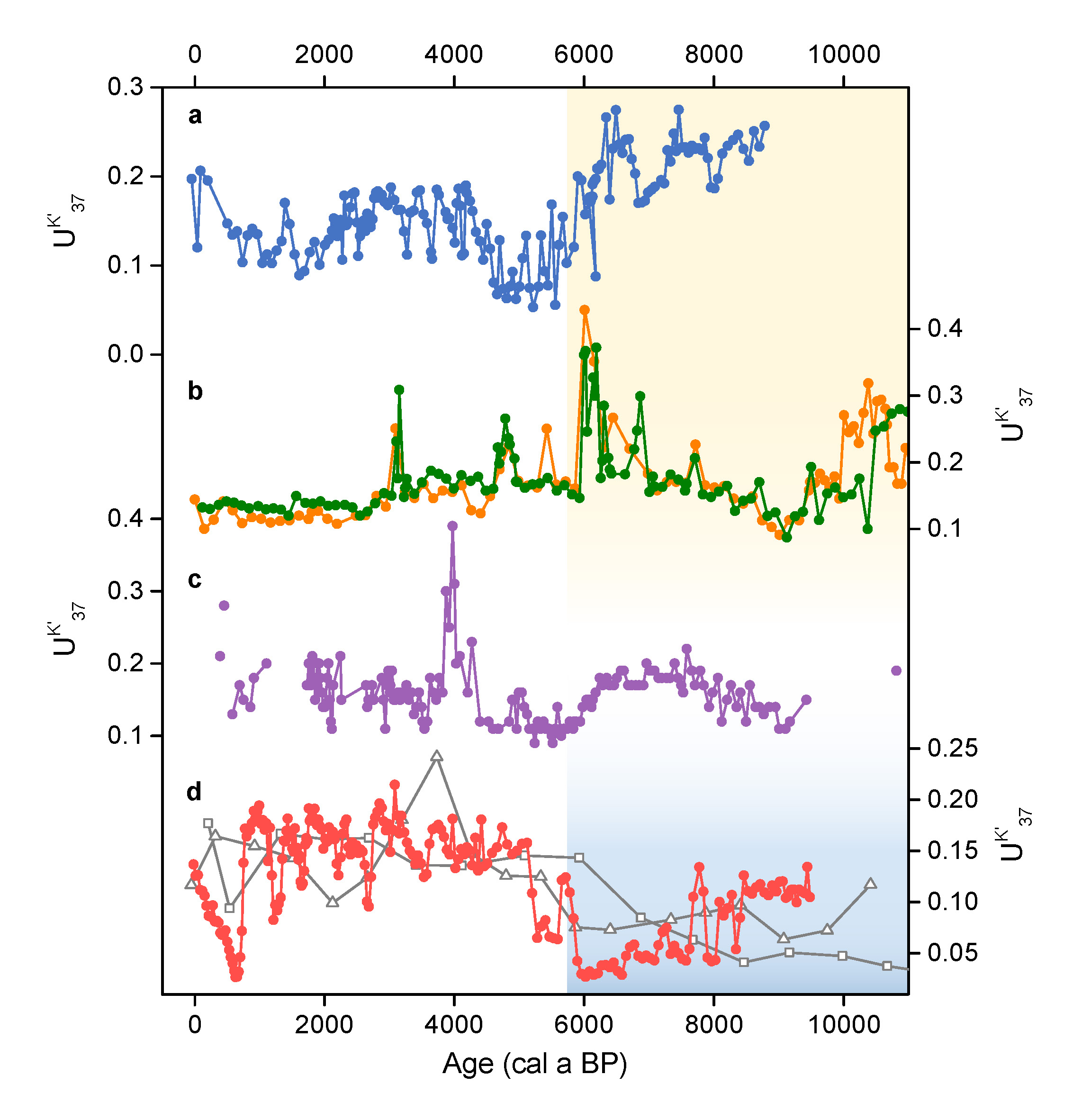


**Supplementary Fig. 5 Alkenone unsaturation (**$\mathbf{U}_{\mathbf{37}}^{\mathbf{K'}}$**) records from mid-latitude Asian lakes.** (**a**) Lake Yihesariwusu (this study), (**b**) Lake Qinghai^18,31,32^, (**c**) Lake Hurleg^18,19^, and (**d**) Lake Ebeyty, Kuchuk, and Maloye Yarovoye (dots, squares, and triangles, respectively, this study). Yellow/blue bar indicates warmer/colder conditions during the early to mid-Holocene.
