# Supplementary material for: Spatial patterns of Holocene temperature changes over mid-latitude Eurasia
Source: Nat Commun. 2024 Feb 19;15:1507. doi: 10.1038/s41467-024-45883-y (PMC10876552; doi:10.1038/s41467-024-45883-y)
Supplement: Supplementary file 3 — Description of Additional Supplementary Files [file 41467_2024_45883_MOESM3_ESM.pdf]

Description of Additional Supplementary Files:

Supplementary Data 1: Alkenone records from mid-latitude Asian lakes and AMS 14C dates from Lake Yihesariwusu and Ebeyty.

Supplementary Data 2: Lists of existing records utilized in this study.
